# Environmental microbial reservoir influences the bacterial communities associated with *Hydra oligactis*

**DOI:** 10.1038/s41598-024-82944-0

**Published:** 2024-12-31

**Authors:** Jay Bathia, Máté Miklós, István Gyulai, Sebastian Fraune, Jácint Tökölyi

**Affiliations:** 1 Institute of Zoology and Organismic Interactions, Heinrich-Heine University, Düsseldorf, Germany; 2 Institute of Evolution, HUN-REN Centre for Ecological Research, Budapest, Hungary; 3 Centre for Eco-Epidemiology, National Laboratory for Health Security, Budapest, Hungary; 4 National Laboratory for Water Science and Water Security, Department of Hydrobiology, University of Debrecen, Debrecen, Hungary; 5 MTA-DE “Momentum” Ecology, Evolution & Developmental Biology Research Group, Dept. of Evolutionary Zoology, University of Debrecen, Debrecen, Hungary

**Keywords:** Ecology, Community ecology, Microbial ecology, Microbial communities, Microbial ecology

## Abstract

The objective to study the influence of microbiome on host fitness is frequently constrained by spatial and temporal variability of microbial communities. In particular, the environment serves as a dynamic reservoir of microbes that provides potential colonizers for animal microbiomes. In this study, we analyzed the microbiome of *Hydra oligactis* and corresponding water samples from 15 Hungarian lakes to reveal the contribution of environmental microbiota on host microbiome. Correlation analyses and neutral modeling revealed that differences in *Hydra* microbiota are associated with differences in environmental microbiota. To further investigate the influence of environmental bacterial community on the host microbiome, field-collected *Hydra* polyps from three populations were cultured in native water or foreign water. Our results show that lake water bacteria significantly contribute to *Hydra* microbial communities, but the compositional profile remained stable when cultured in different water sources. Longitudinal analysis of the in vitro experiment revealed a site-specific change in microbiome that correlated with the source water quality. Taken together, our findings demonstrate that while freshwater serves as a critical microbial reservoir, *Hydra* microbial communities exhibit remarkable resilience to environmental changes maintaining stability despite potential invasion. This dual approach highlights the complex interplay between environmental reservoirs and host microbiome integrity.

**Supplementary Information:**

The online version contains supplementary material available at 10.1038/s41598-024-82944-0.

## Introduction

The complex interactions between metazoans and their associated bacterial communities have been extensively studied, revealing significant impacts on various aspects of host fitness, including development and growth rate, metabolism, reproduction, aging and survival^1–6^. Understanding these fitness effects is one of the key aims of microbiota research, but is hampered by the substantial variability of microbial communities in space^7–13^ and time^14–17^, which makes it difficult to identify key bacterial players with a causal effect in determining host fitness.

The large variation of microbial communities raises the question how these communities are assembled and maintained, given their often-crucial effects on host fitness. Early studies stressed the importance of vertically inherited microbial partners that are passed on from generation to generation, resulting in co-evolution between hosts and symbionts (a phenomenon termed phylosymbiosis;^18–22^). A key prediction of the phylosymbiosis hypothesis is that microbial communities will be more similar within members of a host taxonomic group compared to those outside the group. Such phylogenetic patterns have been detected in a range of animal groups from Cnidarians to Mammals^22–28^.

On the other hand, it is clear that host-associated microbial communities, even if they are inherited vertically from parents, are not isolated from the external environment and the microorganisms that live around them. As a consequence, environmental microbiota can invade and shape host-associated microbial communities, resulting in increased environmental signals and reduced phylogenetic patterns in the composition of host associated bacterial communities. For instance, in fish and shrimp grown in aquacultures, changes in the bacterial community of the water are often highly correlated with differences in the gut bacterial community (reviewed in^29^). In fishes, shrimps and corals, there is a substantial overlap between bacterial taxa found in animals and their surrounding environment^29–31^. Likewise, among amphibians, culturing tadpoles in a different environment (water sourced from another lake) results in altered microbial community composition^32^. Among freshwater zooplankton, the signature of host species identity seems to be weak^33^, while in another group of freshwater invertebrates (*Hydra*), the species signature is present but is substantially molded by the identity of the environment^13^. A phylogenetic signal is entirely lacking, or is weak, in a large number of animal groups investigated thus far^19,34–37^.

A current view emerging from these complex ecological patterns is that host-associated microbial communities are initially established through maternal/inherited factors, and later molded by the inflow of bacterial species from other individuals and the outside environment (called leaky vertical transmission or mixed mode of transmission;^38–40^). For instance, in cnidarians, embryonic stages are associated with well-defined microbial taxa that are either established by antimicrobial peptides synthesized by the mother^41^ or by specific organic materials produced by itself to provide resources for beneficial microbes^42^. These microbial communities are subsequently being altered through stochastic losses and gains from the environment. Conversely, in many species neonates are largely devoid of bacteria, and microbiota communities develop later during development from environmental inocula^43,44^, parental sources^45–49^, or through inter-host dispersal^50^. However, the ecological processes of bacterial species gain and losses from environmental sources are still poorly understood in most species.

Freshwater *Hydra* are well-known for their dependence on the microbiome for normal development and tissue homeostasis^51,52^, physiological performance^52–54^ and relationship to pathogens^55^. Early studies showed that *Hydra* species harbor distinct, species-specific microbial communities that persist in the long-term, even if these species are cultured under identical conditions in the laboratory^24,55^. More detailed investigations, however, revealed that in addition to these species-specific signatures, population identity contributes substantially to variation in microbiota composition^13^, suggesting that environmental sources might contribute to microbiota assembly in *Hydra*.

Here we aimed to quantify the contribution of environmental microbiota to host-associated bacterial communities in *Hydra* using both correlative and experimental methods. To this end, we sampled multiple sites within 15 *Hydra* populations in Hungary and sampled both polyps and the water to identify microbial communities and estimate the similarity and overlap between polyp and water samples. Next, we used polyps from a subset of these populations to experimentally alter their microbial environment by culturing them in lake water from a different population. Our results show that bacteria from lake water can contribute to host-associated microbial communities, but they do not abruptly shift *Hydra* microbiota. Hence, polyps are colonized by bacterial communities that are relatively resistant to invasion from environmental sources.

## Methods

### Field sampling

To test the correlation between the composition of water and *Hydra*-associated microbial communities, field sampling of *Hydra* polyps and water samples was conducted during November–December 2020 at 15 water bodies in Hungary (Table 1). Within each population, *Hydra* and water samples were obtained from three discrete sites along the shoreline, each situated at least 10 m apart. First, we searched the submerged vegetation for *Hydra* polyps by placing pieces of aquatic vegetation into sterilized plastic boxes. A minimum of three polyps were collected from each site, carefully removed from the vegetation using an automatic pipette with sterile tips, and individually transferred into sterile Eppendorf-tubes filled with lake water for transport to the laboratory. Simultaneously, water samples were collected in 1-liter sterile bottles from the exact locations where polyps were discovered. Both the *Hydra* polyps and water samples were promptly transported to the laboratory on the day of collection in a cooled box.

In the laboratory, polyps were checked for species identity under a stereomicroscope (Euromex Stereo Blue). This examination was carried out within the Eppendorf-tube, to prevent the introduction of foreign bacteria. Several *Hydra* species co-occur in Hungary, however, the most abundant species during the time of collection was *Hydra oligactis*, a cold-adapted species that reaches peak population sizes during the winter (see^56^ for a description of the species life cycle). Consequently, our sample collection was confined to *H. oligactis*, and polyps clearly belonging to other species were discarded. However, some polyps (especially smaller ones) cannot be identified based on morphological cues alone and we used an additional, molecular method to verify species identity after DNA extraction from polyps (described in section ***DNA extraction***,*** PCR-based species identification and sequencing***). Following morphological species identification, polyps were gently washed with sterile-filtered lake water 3 times under a sterile hood to eliminate adhering debris, and then transferred to empty sterile Eppendorf-tube. Finally, they were frozen overnight at − 20 °C before DNA extraction (see below).

Water samples were filtered through a 0.22 μm PES membrane filter (FilterBio^®^, Nantong, P. R. C.) and the filters were subsequently utilized for DNA extraction. Due to the presence of substantial suspended material in most samples, only 100–200 ml were used for the filtration process.

## Water exchange experiment

To experimentally investigate the influence of water as a bacterial source in shaping *Hydra* microbiota assembly, we conducted water exchange experiments in the laboratory with field-collected polyps and lake water. In November 2021, polyp samples were collected from three distinct populations: M26, M52 and M109 (refer to Supplementary Table 1 for site descriptions). Corresponding water samples were also collected from these sites (henceforth called “native water”). In addition, lake water was collected from three additional water bodies (M79, M25 and M28) to culture *Hydra* polyps in different environments (henceforth called “foreign water”). These latter populations were chosen to mirror the characteristics of the original sites in terms of habitat. Specifically, location M79 (Tiszavalk, 47.69003 N; 20.7434E) represented a canalized river similar to location M26; location M25 (see Supplementary Table 1) represented a large fishing lake with sparse shoreline vegetation similar to location M52, while location M28 represented a small oxbow lake with dense shoreline vegetation akin to lake M109. These water bodies were further characterized based on a number of chemical parameters (see section “Water quality measurements” below).

Both polyps and water were collected using sterile equipment and promptly transported to the laboratory on the day of collection. In the laboratory, polyps from each of the three populations were randomly assigned to two groups: one group cultured in native-water and another group cultured in foreign water (matched based on habitat characteristics: M26 with M79, M52 with M25, M109 with M28), resulting in a total of six groups. Each group comprised five jars filled with 200 ml of lake water (either from the same population or a different one). Prior to use, lake water underwent filtration through a paper filter (porosity: 8 μm) to remove debris and zooplankton while retaining microorganisms. Initially, 10 polyps were introduced into each jar and maintained for four weeks. Every week, one polyp from each jar was removed for DNA isolation and 16S sequencing. Additionally, to establish baseline conditions, five polyps were isolated for DNA extraction on the day of collection.

## DNA extraction, PCR-based species identification & sequencing

DNA was extracted through a chloroform-isoamyl alcohol extraction method; detailed description of the protocol can be found in the supplementary of^57^.

To check that only *H. oligactis* samples were included in our sample, host species identity was verified using PCR reactions with the extracted DNA as a template. This method relies on amplification of DNA sequences with species-specific primer pairs. Two sets of primers were used: HyO_T_MSAT_001 (fw: CTAGACTTACTGCGTCGCCC; rv: AGCGTGTCTCGAAATCATACA) and HyO_T_MSAT_002 (fw: ACTGGTAGATAAGTCTGGTGATGA; rv: TCGAATAACGATCCGAGACATCT). The first of these gives a PCR product for *H. vulgaris* and *H. oligactis*, while the second one only for *H. oligactis*. In the case of a third, morphologically similar *Hydra* species co-occurring with the former two *H. circumcincta*, neither primer gives a PCR product. PCRs were conducted with the following cycling conditions : 96 °C, 3 min; 35 × (96 °C, 45 s; 62 °C, 30 s; 72 °C, 90 s); 72 °C, 10 min; 18 °C, infinity. PCR products were checked on 1% agarose gel with 0.007% GelRed (Biotium, Fremont USA).

The 16S rRNA gene was amplified using uniquely bar-coded primers flanking the V1 and V2 hypervariable region (27 F–338R) with fused MiSeq adapters and heterogeneity spacers in a 25-µl PCR^58^. For the traditional one-step PCR protocol, we used 4 µl of each forward and reverse primer (0.28 µM), 0.5 µl dNTPs (200 µM each), 0.25 µl Phusion Hot Start II High-Fidelity DNA Polymerase (0.5 Us), 5 µl of HF buffer (Thermo Fisher Scientific, Inc., Waltham, MA, United States), and 1 µl of undiluted DNA. PCRs were conducted with the following cycling conditions: 98 °C, 30 s; 30 × (98 °C, 9 s; 55 °C, 60 s; 72 °C, 90 s); 72 °C, 10 min; 10 °C, infinity; and checked on a 1.5% agarose gel. The concentration of the amplicons was estimated using a GelDoc™XR + System coupled with Image Lab™Software (BioRad, Hercules, CA, United States) with 3 µl of O’GeneRulerTM100 bp Plus DNA Ladder (Thermo Fisher Scientific, Inc., Waltham, MA, United States) as the internal standard for band intensity measurement. The samples of individual gels were pooled into approximately equimolar sub-pools as indicated by band intensity and measured with the Qubit dsDNA br Assay Kit (Life Technologies GmbH, Darmstadt, Germany). Sub-pools were mixed in an equimolar fashion and stored at − 20 °C until sequencing.

Library preparation for shotgun sequencing was performed using the NexteraXT kit (Illumina) for fragmentation and multiplexing of input DNA following the manufacturer’s instructions. Amplicon sequencing was performed on the Illumina MiSeq platform with v3 chemistry (2 × 300 cycle kit).

## Water quality measurements

To further characterize the six water bodies that were used in the water exchange experiment, we performed measurements on the chemical properties of water at three distinct timepoints spanning almost a complete year: 13th July 2021, 8th November 2021 and 22nd April 2022. Non-filtered water samples were collected from the top (50 cm) surface of the water and brought to the laboratory within the same day. Three distinct water samples were collected from each of the six locations from distinct locations that were at least 10 m apart. These water samples were analyzed for chemical variables, as follows. Total suspended solids and total dissolved solids (TSS and TDS, mg/l), which were measured according to the Hungarian Standard MSZ 260-3:1973; chloride-ions (Cl^−^, mg/l) which were determined using precipitation titration, as described in^59^; total-phosphorus (mg/l) was measured according to the Hungarian Standard MSZ 12750-17:1974; nitrate-nitrogen (NO3^−^-N, mg/l), nitrite-nitrogen (NO2^−^-N, mg/l), ammonium-ions (NH4^+^, mg/l) were measured according to the Hungarian Standards MSZ (12750-16:1974, 1484-13:2009, 7150-1:1992, and 260 − 12:1987); sulfate ions (SO_4_^2−^, mg/l) were measured according to the International Standard ISO 15923-1:2013; permanganate-based chemical oxygen demand (CODsMn, mg/l O_2_) was measured according to the Hungarian Standards MSZ (448 − 20:1990, and ISO 6060: 1991); hydrogen carbonate (HCO_3_^−^) content measurement was based on the Hungarian Standard MSZ 448 − 11:1986. Finally, chlorophyll-a content was measured using hot methanol extraction and spectrophotometry^60^. Spectrophotometric measurements were performed with a Hach Lange DR6000™ one-way UV-VIS spectrophotometer.

Water quality data were analyzed by employing Principal Components Analysis (PCA), performed separately for the three sampling times.

## Sequence processing and data analyses

The 16S amplicon data were analyzed using a standardized QIIME2 (version- 2021.11) pipeline^61^. The sequence qualities were checked using FastQC. The sequence adapters were trimmed using CutAdapt. The reads were trimmed by 7 bp from left and 21 bp from right to achieve the minimum quality score 10. Further, the sequences were processed using DADA2 ^62^ for chimera removal and get the amplicon sequence variants (ASVs) for each sample. A further filter was applied to remove the ASVs that had a total count of < 50 in the entire dataset. For taxonomic annotation, we used SILVA 138.1 as our reference dataset^63^. It was trained for the V1-V2 region and for the sequencing primers used by the sequencing facility (F- 5’-AGRGTTYGATYMTGGCTCAG-3’ and R- 5’-TGCTGCCTCCCGTAGGAGT-3’) with the help of RESCRIPt plugin^64^. The reference reads with minimum and maximum lengths of 100 and 400 bp respectively were retained. It was further quality controlled by dereplication. For alpha diversity analysis, the samples were rarified to the minimum number of reads for a given experiment. The beta-diversity visualization plots were generated using the R package MicrobiomeStat^65^ by importing the distance matrices generated by QIIME2 into R (version- 2023.03.1) using qiime2R^66^. To study the effect on change in microbiome alpha and beta diversity along the four weeks, we utilized the “qiime-longitudinal-analysis” function. We calculated the statistical significance of group beta dissimilarity using the PERMANOVA function that returned p-value based on 1000 permutations. These p-values were then adjusted for false discovery rate (FDR) using Benjamini-Hochberg method. The differential taxa abundance was calculated using the ANCOMBC function in QIIME2; the significance level was set to alpha < 0.05 and minimum fold change of 2.

To explore the relationship between water microbiota and *Hydra*-associated microbiota, we computed pairwise distances between water and polyp samples employing four metrics: Bray-Curtis, Jaccard presence-absence, Weighted Unifrac and Unweighted Unifrac. First, we utilized these distance matrices to evaluate the effect of sample type (i.e. water vs. polyp sample), population identity and their interaction on microbiome β-diversity, employing the *adonis2* function in the *vegan* R-package *v2.6-4*^67^.

Subsequently, we utilized these distance matrices to conduct a matrix correlation analysis between distance of water samples and distance of polyp samples, employing a Mantel test implemented in the *ape* R package *v5.7-1*^68^. Given the presence of multiple polyp samples for each site, we averaged the distances of polyps from paired sites. Our null assumption posited that the beta-diversity distance of microbial communities in water should not correlate with the beta-diversity distance of microbial communities in polyps if the latter do not harbor bacteria originating from the surrounding environment.

To gain additional insight into the assembly of *Hydra*-associated microbial communities, we calculated the Sloan neutral model of community dynamics. This model examines the relationship between two prevalence/abundance metrics of each bacterial taxon: the overall rate of occurrence and mean abundance across polyp samples. The null assumption of this model is that the relationship between occurrence and abundance should adhere to a logistic curve if dispersal between individuals is entirely determined by neutral processes. Essentially, bacterial taxa that are more abundant on polyps should be more likely to colonize novel hosts, assuming all other factors are equal^69,70^. Departures from this pattern suggest non-neutral processes; for instance, taxa under-represented on hosts could be actively removed by the host immune system or by its symbiotic microbial communities. Conversely, over-represented taxa might be abundant species in the environment that end up on *Hydra* but fail to establish large population sizes there. To test this hypothesis, we correlated departures from neutrality, calculated as the residuals of the binomial Generalized Linear Model representing the Sloan Neutral Model on one hand, and abundance or occurrence of taxa in water samples on the other. The null assumption for this relationship was that if water bacteria are not overrepresented on polyps, then there should be no significant correlation between these variables. The Sloan Neutral Model was computed from genus-level ASV data. Genera with a residual greater than the 97.5% percentile were considered overrepresented taxa, while those with the residual less than the 0.25% percentile were considered underrepresented^69^.

To understand patterns of co-associations among bacterial taxa in our samples, we employed the NetCoMi package in R^71^ to construct statistical microbial association networks from abundance data. We selected the top 30 bacterial genera from both the water and polyp samples. Using their abundance data, a co-occurrence network was built utilizing SPRING^72^, a semi-parametric rank-based method that estimates correlation between taxa abundances while naturally dealing with excess zeros characteristic for microbial abundance data. The bacterial clusters identified in this way were visualized with the *ggraph v2.2.1*^73^.

## Results

### Microbiome differences on *Hydra* are associated with differences in water microbiome

A total of *N* = 232 samples were successfully sequenced for the correlative study, of these, *N* = 40 were water samples and *N* = 192 were polyp samples. Five water samples failed to be sequenced, resulting in an average of 2.7 water samples per population (two populations, M52 and M38, had just a single water sample successfully sequenced while in population M25 two water samples were successfully sequenced). The average number of polyp samples successfully collected and sequenced was 12.6 per population (range: 11–16).

**Fig. 1:**
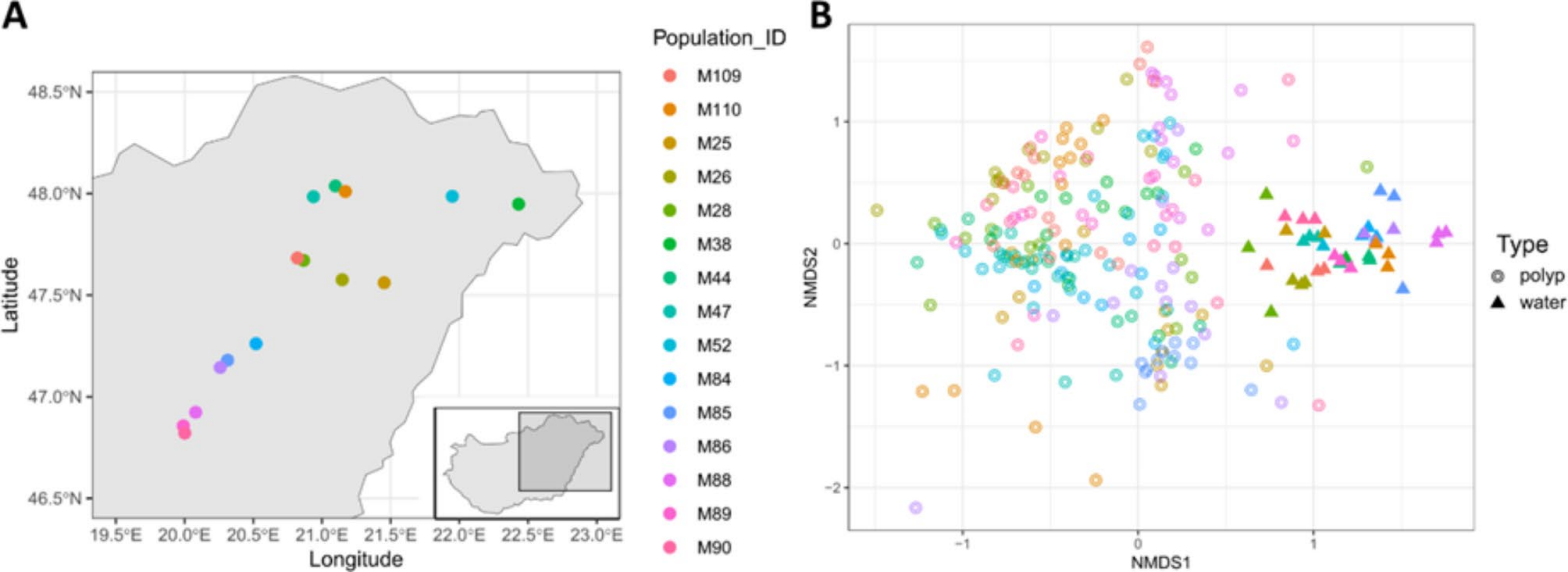
**(A)** Map representing locations of sample collection sites in Hungary (full map in inset—map created using ‘eurostat’ R package, version 4.0.0.9003^101^). **(B)** Non-metric multidimensional scaling (NMDS) analysis based on Bray-Curtis distances of microbial communities found on *Hydra* polyps and matching environmental (water) samples.

The Adonis analysis performed on the Bray-Curtis dissimilarity revealed a significant separation of water and *Hydra* microbiome composition (F_1,202_=68.03, R^2^ = 0.18, *p* < 0.001), a significant difference between populations (F_14,202_=5.45, R^2^ = 0.20, *p* < 0.001) and a significant interaction between sample type and population (F_14,202_=2.79, R^2^ = 0.10, *p* < 0.001; Fig. 1). Other β-diversity indices resulted in the same results (see Supplementary Fig. 1).

**Fig. 2:**
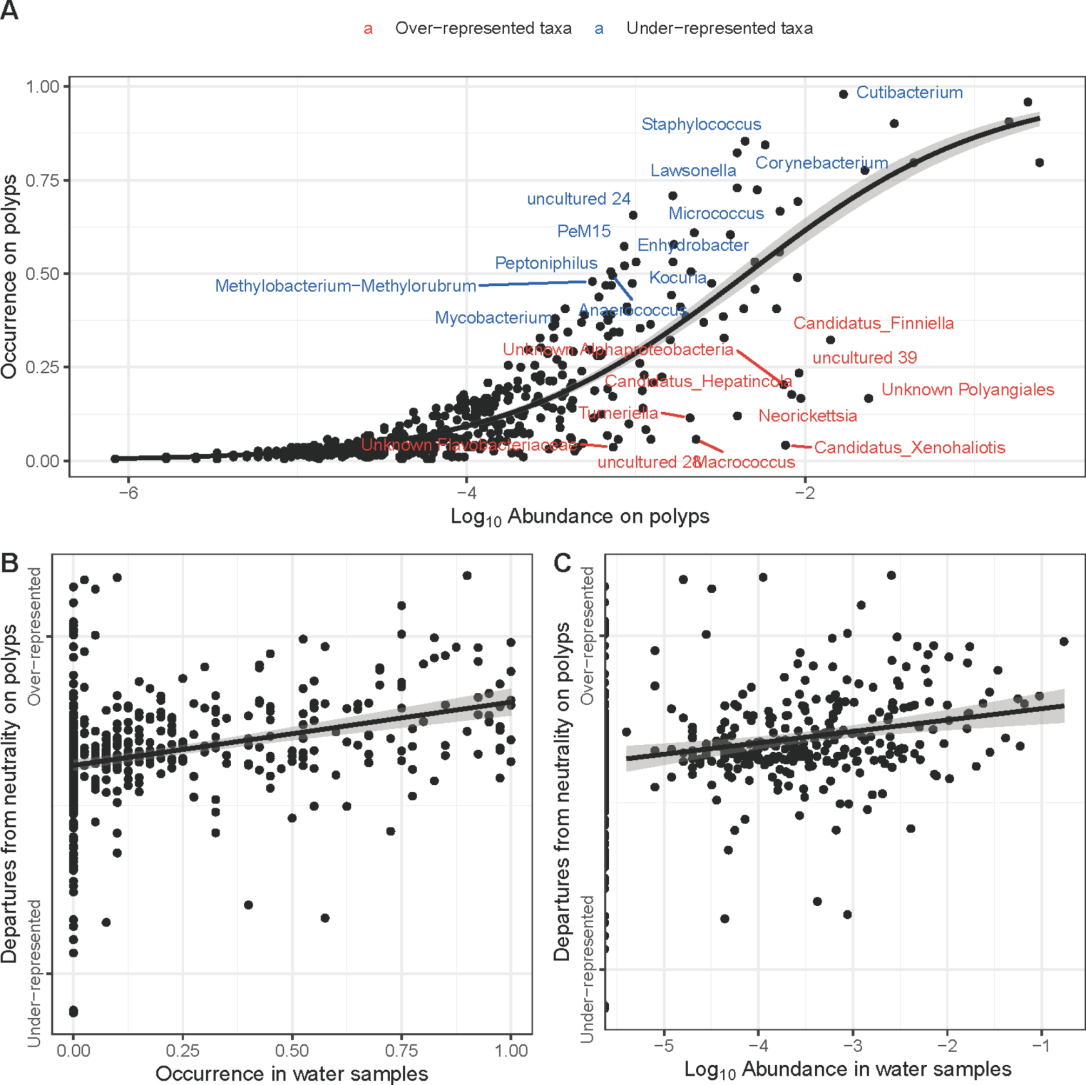
(**A**) Sloan neutral model showing the occurrence of microbial taxa within the polyps samples as a function of their mean abundance. Taxa that are close to the regression curve are assumed to follow a neutral process of dispersal from polyp to polyp, those with negative residuals are underrepresented on polyps, while those with positive residuals are overrepresented. Panels (**B**) and (**C**) show that overrepresented taxa on polyps are more likely to have a higher abundance and occurrence in water samples.

Sloan Neutral Model calculations were used to identify over- and under-represented bacterial genera associated with *Hydra*. While the top 10 over-represented bacterial genera were: *Corynebacterium*, *Cutibacterium*, *Kocuria*, *Lawsonella*, *Micrococcus*, *Methylobacterium-Methylorubrum*, *PeM15* (Actinobacteria), *Peptinophilus*,* Staphylococcus*, and an uncultured *Rhizobiales Incertae Sedis* (Fig. 2A), the top 10 under-represented bacterial genera were *Candidatus Finniella*, *Candidatus Hepatincola*, *Candidatus Xenohaliotis*, *Macrococcus*, *Neorickettsia*, *Rickettsia*, an unknown Alphaproteobacteria, an unknown Elsteraceae, an unknown Polyangiales and an unknown Rickettsiales (Fig. 2A). Departures from neutrality (i.e. residuals from the Sloan Neutral Model) correlated positively with both occurrence (Spearman’s rho = 0.41, *p* < 0.001; Fig. 2B) and abundance in water samples (Spearman’s rho = 0.40, *p* < 0.001; Fig. 2C), such that bacterial genera that were more abundant in water samples and/or occurred at a higher frequency, were more likely to be over-represented on polyps (or vice versa).

The Mantel test revealed a significant correlation between distance of water samples and distance of polyp samples (Jaccard presence-absence: z = 367.3; *p* = 0.005; Bray-Curtis: Z = 299.0, *p* = 0.009; unweighted UniFrac: z = 250.8, *p* = 0.019; weighted UniFrac: z = 129.6, *p* = 0.063).

**Fig. 3:**
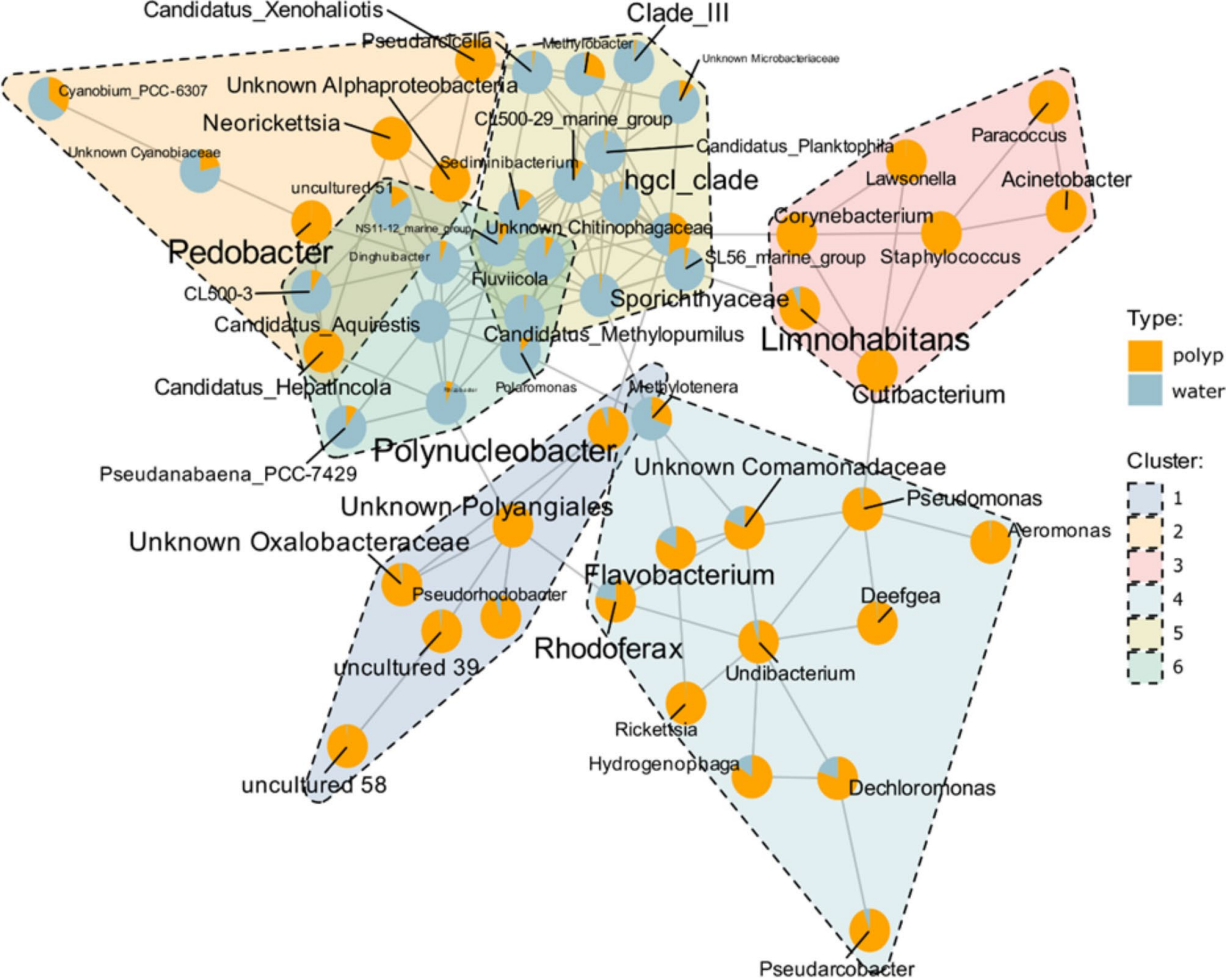
Association network among top bacterial taxa found on polyps and water. Bacterial clusters are shown with different colors. The pie charts show total abundance in polyps vs. water samples, while label sizes are proportional to overall abundance.

Investigation of the patterns of bacterial co-occurrence of top bacterial genera in water and polyp samples revealed six distinct bacterial clusters (Fig. 3). Some of these clusters included common members of *Hydra*-associated microbial communities. For instance, Cluster 4 included, among others, the bacterial genera *Rhodoferax*, *Pseudomonas*, *Aeromonas and Undibacterium*, taxa that form stable associations with *Hydra* in laboratory studies^51,55,74,75^, while Cluster 1 included *Polynucleobacter*, also known to form stable co-associations with *Hydra*^41,76^. Cluster 3 consisted of bacterial genera that were much less abundant in water than in polyps (e.g. *Corynebacterium*, *Cutibacterium*, *Lawsonella*, *Micrococcus*, *Staphylococcus*), while Cluster 2 contained both polyp-specific taxa (e.g. *Pedobacter*) and water-specific genera (e.g. *Cyanobium*). The final two clusters (Clusters 5 and 6) contained taxa that were more abundant in water samples (e.g. the hgcI clade (Actinobacteria), Clade III of SAR11, *CL500-3* (Planctomycetes), *Fluviicola* and *Polaromonas*, among others).

## The microbiome shows resilience to colonization by foreign bacteria

Water quality measurements from the six sites involved in the water exchange experiment were analyzed using PCA. On all three sampling occasions, the first two principal components explained ~ 75% of the total variation (Supplementary Fig. 2). Ammonium, nitrite and nitrate concentrations largely covaried with each other, just as Carbonate, Sulphate, Phosphate and Total Dissolved Solids. Members of the three pairs of populations showed distinct chemical properties, at least in some seasons. The largest difference was found between the chemical properties of water from populations M26 and M79, which differed from each other in all seasons. The chemical properties of water from populations M52 and M25 differed in all seasons, except the summer. Finally, populations M28 and M109 were the most similar to each other, although their chemical properties differed during the summer (Supplementary Fig. 2).

**Fig. 4:**
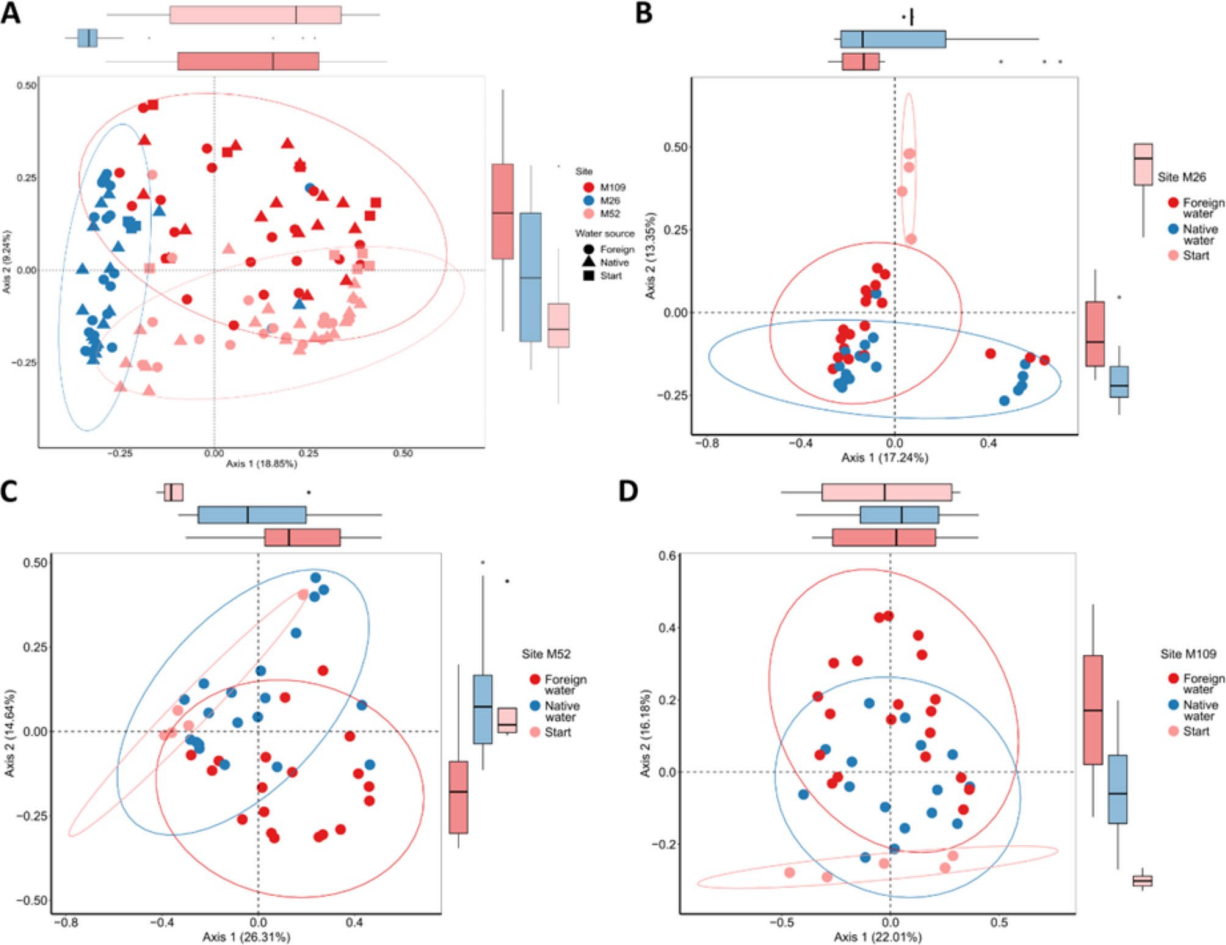
Beta diversity PCoA plots showing Bray-Curtis dissimilarity of polyps from different sites that were maintained in the lab with water from ‘native’ or ‘foreign’ source. (**A**) The PCoA plot shows dissimilarity among the microbiome of polyps from the three sites (ADONIS, R^2^ = 0.190758, *p* = 0.001) (**B**–**D**) There was a clear separation between the start samples and the samples receiving either of the waters cumulatively (Bray-Curtis dissimilarity, PERMANOVA: M26- start vs. native—adj.*p* < 0.05, start vs. foreign—adj.*p* < 0.05; M52- start vs. native—adj.*p* < 0.05, start vs. foreign—adj.*p* < 0.05; M109- start vs. native—adj.*p* < 0.05, start vs. foreign—adj.*p* < 0.05).

Polyps from three sites (M26, M52 and M109) were thus incubated in the lab with lake water from the same (native) or different (foreign: M79, M25 and M28 respectively) sites and sampled after 1, 2, 3 and 4 weeks of incubation (foreign water sources for the corresponding sites are mentioned in materials and methods). Beta diversity analysis showed a clear difference between the 3 populations (Bray-curtis dissimilarity PERMANOVA adj. p-value < 0.01 and Jaccard distances pairwise comparison: PERMANOVA adj. p-value < 0.01 for all sites) (Fig. 4A, supplementary Fig. 5A and supplementary Table 2), irrespective of whether they received the water from the ‘native’ lake or ‘foreign’ lake. This indicates that each population maintains a unique microbiome composition.

### Incubation with native/foreign water influences beta- but not alpha-diversity

**Fig. 5:**
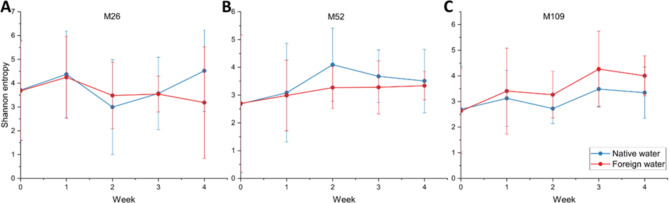
Alpha diversity of the site-specific samples along the 4 weeks depending upon the source of cultivation water. The graphs represent the changes in Shannon entropy for sites (**A**) M25, (**B**) M52 and (**C**) M109. There was no significant change in alpha diversity for all sites at any time point.

To get a deeper insight into temporal changes, we segregated the samples into individual sites and compared different alpha and beta diversity metrics between the samples receiving native or foreign water. For all sites, there was a clear shift in the combined beta-diversity for all time points (week 1–4) as compared to the start point (Fig. 4B, C,D and supplementary Fig. 5-B, C,D). However, there was no difference in the alpha diversities between the samples receiving native or foreign water at any time point as well as no difference compared to the starting point (Fig. 5, Supplementary Fig. 4). These results indicate the gain and loss of certain taxa without affecting the community’s richness.

### Water source did not affect the abundance ß-diversity but only differential presence

**Fig. 6:**
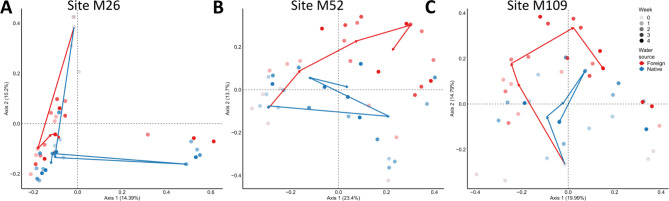
Beta diversity PCoA plots showing Jaccard index for sites (**A**) M26, (**B**) M52 and (**C**) M109 that were maintained in the lab with water from ‘native’ or ‘foreign’ source. The time points from start (0 week) to 4 weeks are shown with increasing color intensity. The arrows indicate the average shift in diversity at a given time point.

The beta-diversity showed an interesting change in diversity with time. We could clearly observe a population-specific alteration in microbiome along the 4 weeks (Fig. 6, Supplementary Fig. 6), indicating a gain and loss of microbial members over time depending upon the water source.

For all sites, both native as well as foreign water treated animals, showed deviations from the starting time point as apparent on Bray-Curtis-dissimilarity (considers abundance of ASVs) and Jaccard-index (considers only presence-absence of ASVs) beta diversity metrics (Fig. 4, supplementary Fig. 4–B, C,D; Supplementary Tables 3–5). The only exceptions were the samples from site M109 that showed minor or no change in Bray-Curtis dissimilarity for native water source compared to the start point (Supplementary Table 5).

**Fig. 7:**
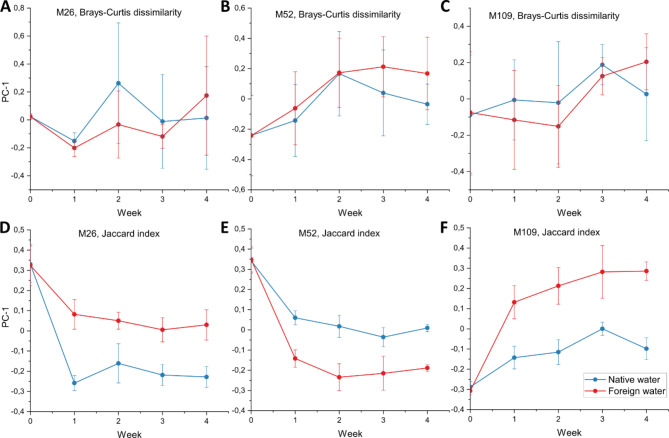
Beta volatility plots for all three sites along the time scale. The plots represent the variation observed on the first axis of the PCoA plots (PC-1) for each population between the two treatments.

For all sites, there was a significant difference observed for presence-absence (Jaccard) index (Tables 1, 2 and 3) between native and foreign water treatments indicating a gain or loss of taxa upon treatment. In addition, we employed longitudinal analysis to compare the temporal variation in beta-diversity. A clear separation was observed between both the treatments in Jaccard index (Fig. 7D–F), but not in Bray-Curtis dissimilarity (Fig. 7A–C). It was interesting to note that the major changes in the beta diversity for both the metrics were already visible after the first week of incubation in the lab. The results clearly indicate the rapid acclimation of the animals to the lab environment followed by stable maintenance of the altered microbiome unperturbed by the supply of environmental microbes.

### Changes in the bacterial community are dependent on the foreign water quality

Since there was a clear gain and loss of bacteria as evident by the Jaccard index, we also looked for the taxa that were differentially abundant due to the water treatments in each site. Differential abundance analysis using ANCOMBC^77^ revealed an interesting site-specific pattern of gain and loss of microbes. As expected, the comparison between the polyps receiving native and foreign water revealed the presence of various different taxa (Supplementary Fig. 8). At the end of 4 weeks, samples from all sites for both treatments acquired an ASV of genus *Perlucidibaca* as compared to the start point (Supplementary Fig. 7). Sites M109 acquired considerably more ASVs when cultivated in foreign water as compared to native water indicating the flexibility of microbiota communities of animals from this site to a new environment.

The amount of differential ASVs also correlates with the water quality difference between the native and foreign sources. There was the highest difference in water chemical composition of sites M26 and M79 and had the least amount of differential ASVs in M26 polyps receiving foreign water from M79 (11 differential ASVs). On the other hand, sites M109 and M28 had the most similarity in water quality and polyps from M109 had the highest number of differential ASVs, when incubated in foreign water from M28 compared to start point (59 differential ASVs). Sites M52 and M25 had moderate differences in the water quality and thus there were intermediate numbers of differential ASVs in foreign water treated polyps (32 differential ASVs).

**Table 1:** PERMANOVA results for beta diversity comparisons for site M26.

Group 1	Group 2	Time	Bray-Curtis dissimilarity			Jaccard index		
			Pseudo F	p-value	q-value	Pseudo F	p-value	q-value
Native water	Foreign water	Week 1	3.883862	0.007	0.029455	3.208274	0.013	0.018
		Week 2	1.764576	0.064	0.109714	1.895466	0.009	0.014727
		Week 3	1.193692	0.228	0.293143	2.047804	0.006	0.014727
		Week 4	1.107317	0.201	0.278308	1.852697	0.009	0.014727

Bray-Curtis dissimilarity and Jaccard distance between different groups. q-value represents the corrected p-value with Benjamini & Hochberg correction. Site M26.

**Table 2:** PERMANOVA results for beta diversity comparisons for site M52.

Group 1	Group 2	Time	Bray-Curtis dissimilarity			Jaccard index		
			Pseudo F	p-value	q-value	Pseudo F	p-value	q-value
Native water	Foreign water	Week 1	1.926335	0.095	0.110323	2.959395	0.009	0.014727
		Week 2	4.131159	0.006	0.036	3.070269	0.012	0.014897
		Week 3	2.000821	0.047	0.0705	2.520471	0.012	0.014897
		Week 4	2.260973	0.019	0.048	2.393253	0.01	0.014897

Bray-Curtis dissimilarity and Jaccard distance between different groups. q-value represents the corrected p-value with Benjamini & Hochberg correction. Site M52.

**Table 3:** PERMANOVA results for beta diversity comparisons for site M109.

Group 1	Group 2	Time	Bray-Curtis dissimilarity			Jaccard index		
			Pseudo F	p-value	q-value	Pseudo F	p-value	q-value
Native water	Foreign water	Week 1	2.243876	0.041	0.11	3.702751	0.006	0.024923
		Week 2	1.195352	0.323	0.3912	2.705281	0.008	0.024923
		Week 3	1.865543	0.133	0.217636	2.441059	0.025	0.032143
		Week 4	1.475635	0.238	0.317333	2.499723	0.018	0.027

Bray-Curtis dissimilarity and Jaccard distance between different groups. q-value represents the corrected p-value with Benjamini & Hochberg correction site M109.

## Discussion

In this study, we explored the role of water as a reservoir for shaping the bacterial communities associated with *Hydra*. Our findings indicate that bacteria in lake water can integrate into *Hydra*’s microbiota, albeit to a limited extent, with a relatively small impact on the overall composition of bacterial communities associated with polyps.

Previous studies have investigated the interplay between host-associated and environmental microbiota in aquatic organisms as reviewed in^78^. For instance, studies on frogs have demonstrated that exposing tadpoles or adults to water with increased microbial diversity results in richer skin and/or gut microbial communities^32,79^. Similarly, across various aquatic species, the microbial composition associated with hosts partly mirrors the composition of planktonic microbial communities suggesting that water serves as a reservoir for animal-associated microbiota.

Based on an extensive dataset encompassing 15 *Hydra* populations, our analysis revealed a positive correlation between the dissimilarity of community composition of water samples and those of polyp samples. This suggests that if two water samples collected from distinct sites exhibit greater dissimilarity, the corresponding polyp samples from those sites are also likely to demonstrate increased dissimilarity.

Investigating the distribution of individual bacterial genera across polyp samples, we found that some taxa displayed substantial deviation from a neutral model of distribution, which assumes that patterns of occurrence of bacteria on hosts reflect patterns of overall abundance across individuals^69,70^. Interestingly, taxa that appeared to be overrepresented on polyps (i.e., had a higher occurrence in polyp samples than predicted based on their overall abundance), were significantly more common in water. This suggests that bacteria that are more abundant or more common in water samples could be the ones ending up in the host-associated communities of aquatic animals.

The bacterial taxa found on field-collected polyps formed multiple clusters of co-association. Most of these clusters consisted of bacterial genera that were more abundant on polyps than in water, suggesting that they are core components of *Hydra* microbiota. Among these, several taxa, such as *Rhodoferax*, *Aeromonas* and *Pseudomonas*^51,74^ are known symbionts of *Hydra*. *Polynucleobacter* is a known symbiont that is vertically inherited from *Hydra* mothers to their sexual offspring and is positively associated with *Hydra* fitness^41,76^. *Pedobacter* was another, common bacterial genus on our *Hydra* samples. This genus is mostly found in environmental samples, such as in soil and in freshwater^80^, although some species are components of animal-associated microbiota (e.g. *P. schmidtae* inhabits the guts of planarians^81^).

A somewhat distinct category emerged in Cluster 3. This cluster was composed of bacteria that were much less abundant in water than on polyps and many of them were overrepresented on polyps based on the neutral community dynamics model (i.e., more frequent than expected based on abundance). Taxa included in this group included e.g. *Corynebacterium*, *Cutibacterium*, *Lawsonella*, *Micrococcus*, *Staphylococcus*, bacteria that are commonly associated with animals and often cause disease^82–86^. Their function within *Hydra*-associated microbial communities is currently unclear.

In contrast to the above groups, two clusters contained bacteria that seemed water-specific (i.e., they were more abundant in water than on polyps). The taxa within this group included bacteria such as *Fluviicola*, *Pseudarcicella*, *Polaromonas*, the hgcI clade (Actinobacteria), Clade III of SAR11 and *CL500-3* (Planctomycetes), among many others. *Fluviicola* is a small genus consisting of merely a few species, all of which are environmental species occurring in freshwater, wastewater and soil^87,88^. *Polaromonas* is a genus of extremophiles most abundant in arctic or alpine glaciers, although they are also found within granular activated carbon filters from wastewater treatment facilities^89,90^. *Pseudarcicella* is found within the skin microbiota of freshwater invertebrates (leeches), but also in freshwater environmental samples^91,92^.The groups *hgcI*, *Clade III* and *CL500-3* are all common components of freshwater samples^93–95^. Some of these taxa were also found in a small abundance on *Hydra* polyps. Hence, they might be environmental taxa that end up as components of *Hydra*-associated bacterial communities (even though they might not be permanent members).

The correlative findings detailed above were substantially supported by observations from our laboratory experiment. Specifically, our analysis of beta diversity patterns revealed minimal compositional disparities, as assessed by the Bray-Curtis index, when polyps were cultured in water sourced from foreign habitats compared to their native environments. Conversely, significant differences were noted based on the Jaccard presence-absence index. Consequently, when polyps were cultured in non-native water, certain bacterial taxa vanished while others were introduced into the polyp microbiota, predominantly comprising rare taxa. Similar shifts in green *Hydra* bacterial community is observed upon incubation in water from aposymbiotic animals, predominantly due to acquisition of *Legionella* from the water^53^.

It was interesting to observe that the impact of foreign water varied on the recipient population depending upon the quality of water. The sites with most difference in water quality showed a lower number of differential microbes (site M26 receiving water from site M79) while the sites with the most similarity in water quality showed a higher exchange of microbes (site M109 receiving water from site M28). This indicates that the polyps are more open to exchange of microbes if both the partners are adapted to similar environmental conditions, while the exchange is limited if the host and microbes are adapted to different environments. Microbes coming from the similar environment show pre-adaptation to that environment and would thus be able to better colonize the host from similar environments as documented for human gut associated *Bacteroides fragilis*^96^ where adaptation to gut environment is linked with long-term persistence as a part of microbiome. On the other hand, bacteria coming from a foreign environment cannot colonize the host due to monopolization by the existing pre-adapted microbes^97^. The pre-adaptation phenomenon is also exemplified for green *Hydra* where the invasive and low sugar adapted *Legionella sp. Hvir* from aposymbiotic animals is able to invade but unable to form a stable and long-term relation in a potentially sugar and oxygen rich environment of symbiotic polyps^53^.

The observation that culturing *Hydra* polyps in lake water sourced from different sites does not substantially alter microbiota composition is surprising, as the samples taken from these distinct habitats have distinct microbiota communities^13^. The limited contribution of water-borne bacteria to *Hydra*-associated communities implies that population distinctions may not stem from exposure to distinct bacterioplankton communities. Invasion from alternative sources, such as food or substrate to which *Hydra* are attached, could be plausible alternatives. Population differences might also arise from stochastic shifts in *Hydra*-associated bacterial communities. One such example was visible for all the samples, where the genus *Perlucidibaca* appeared in a high abundance by the end of the experiment. Host genotype effects could be also involved, if we assume that distinct populations are colonized by genetically different *Hydra* populations, each associated with distinct bacteria (as is seen, e.g. in *Nematostella*;^98^). Furthermore, our study was constrained to a single seasonal period (early winter). Colonization from environmental sources may fluctuate throughout the year, potentially increasing during periods of stress, such as heat in the summer. We could also observe site specific alterations in the microbiome composition that can be attributed either to the strain variation of the source populations or difference in invasive species present in the received water. Further investigations are warranted to explore these possibilities.

Interestingly, most of the changes in community composition occurred in the first week of establishment in the lab, and *Hydra* microbiota remained relatively stable thereafter. This observation is especially surprising since the animals were starving during the whole period and likely experienced substantial stress by the end of the experiment. The notable difference in microbiota composition between collected polyps and those observed after one week in the laboratory suggests that transfer to the lab environment influences bacterial dynamics. Similar rapid changes in microbiota composition have been observed in other organisms, such as *Daphnia*, where shifts occurred within one hour of laboratory transfer^99^ and also in symbiotic *Hydra viridissima* upon co-cultivation or water supplement from aposymbiotic animals^53^. In the case of *Hydra oligactis*, such rapid changes could be attributed to factors like limited water movement, absence of natural food sources, or a substrate hosting distinct bacterial communities.

In conclusion, our experiment shows that exposure to different lake water has a detectable effect on the composition of *Hydra* by altering the presence/absence of specific bacteria, without shifting its compositional profile. The experiment was done with animals collected from three distinct populations, with broadly similar results, suggesting that the observed patterns are general for *Hydra*. However, a potential limitation of our experiment could be that the water samples involved in our experiments do not differ too much from each other in bacterial composition, and a stronger restructuring of *Hydra* microbial communities would be observed with more distinct water types. While we were able to establish the differences in the chemical properties of water samples, sequencing of bacterial communities from water samples, native as well as foreign water involved in the experiment, failed. Nonetheless, even if the difference between the lake water bacterioplankton differences were small, they are likely to be ecologically highly relevant, since they mimic e.g., the effects of floods or the mixing of water from nearby populations.

## Electronic supplementary material

Below is the link to the electronic supplementary material.

Supplementary Material 1

## Data Availability

The raw data (FASTQ format) as well as associated metadata from 16S amplicon sequencing has been submitted to NCBI-Sequence Read Archive (SRA) under the BioProject: PRJNA1119384.
