# Supplementary material for: Environmental microbial reservoir influences the bacterial communities associated with Hydra oligactis
Source: Sci Rep. 2024 Dec 31;14:32167. doi: 10.1038/s41598-024-82944-0 (PMC11688501; doi:10.1038/s41598-024-82944-0)
Supplement: Supplementary file 1 — Supplementary Material 1 [file 41598_2024_82944_MOESM1_ESM.pdf]

Environmental microbial reservoir influences the bacterial communities associated with *Hydra oligactis*

Authors: Jay Bathia[1,*], Máté Miklós[2,3], István Gyulai[4], Sebastian Fraune[1] & Jácint Tökölyi[5,*]

Supplementary material:

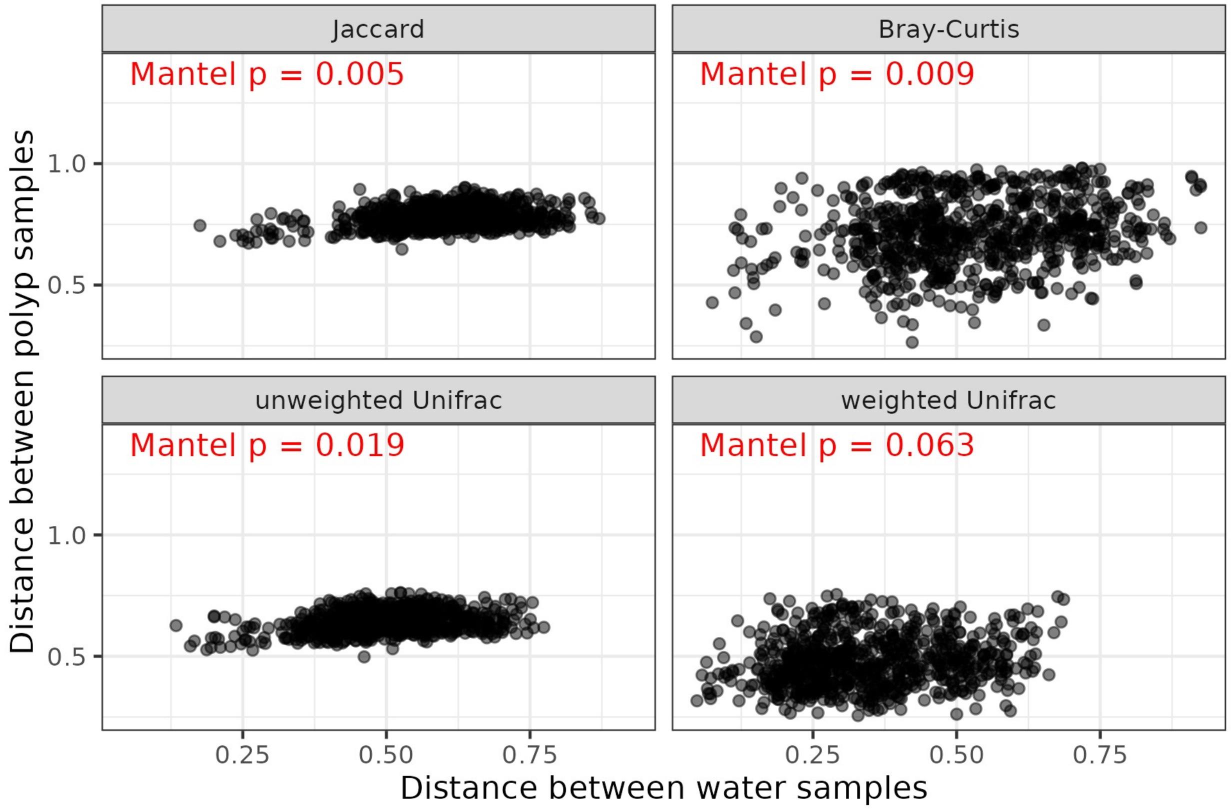

Supplementary figure 1. *Matrix correlation between pairwise distance of water samples and pairwise distance of polyps samples from distinct sites. Four distinct beta diversity metrics were used to estimate the distance between sites. In the case of polyp samples, the distance between multiple individuals was averaged, while no averaging was done for water samples (only a single sample was collected per site).*

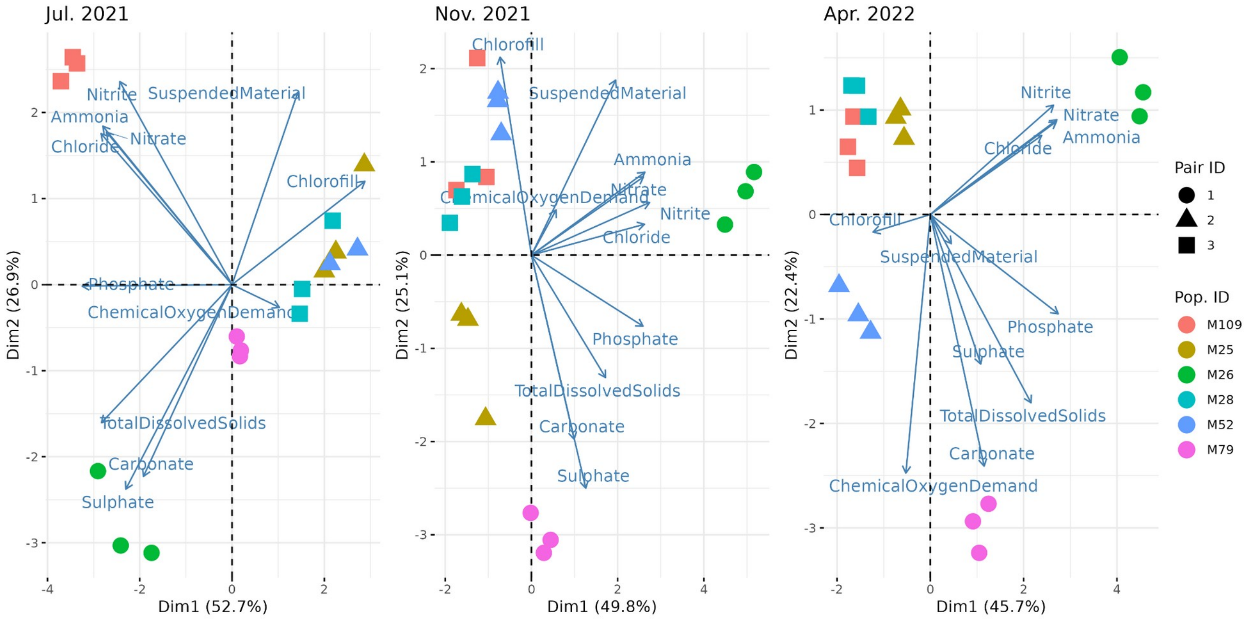

Supplementary figure. 2*: Biplots showing results from the Principal Components Analysis done on the water quality measurements obtained from the six populations involved in the water exchange experiment. Populations are shown by distinct colors and pairs of populations between which water was exchanged are shown by distinct symbols.*

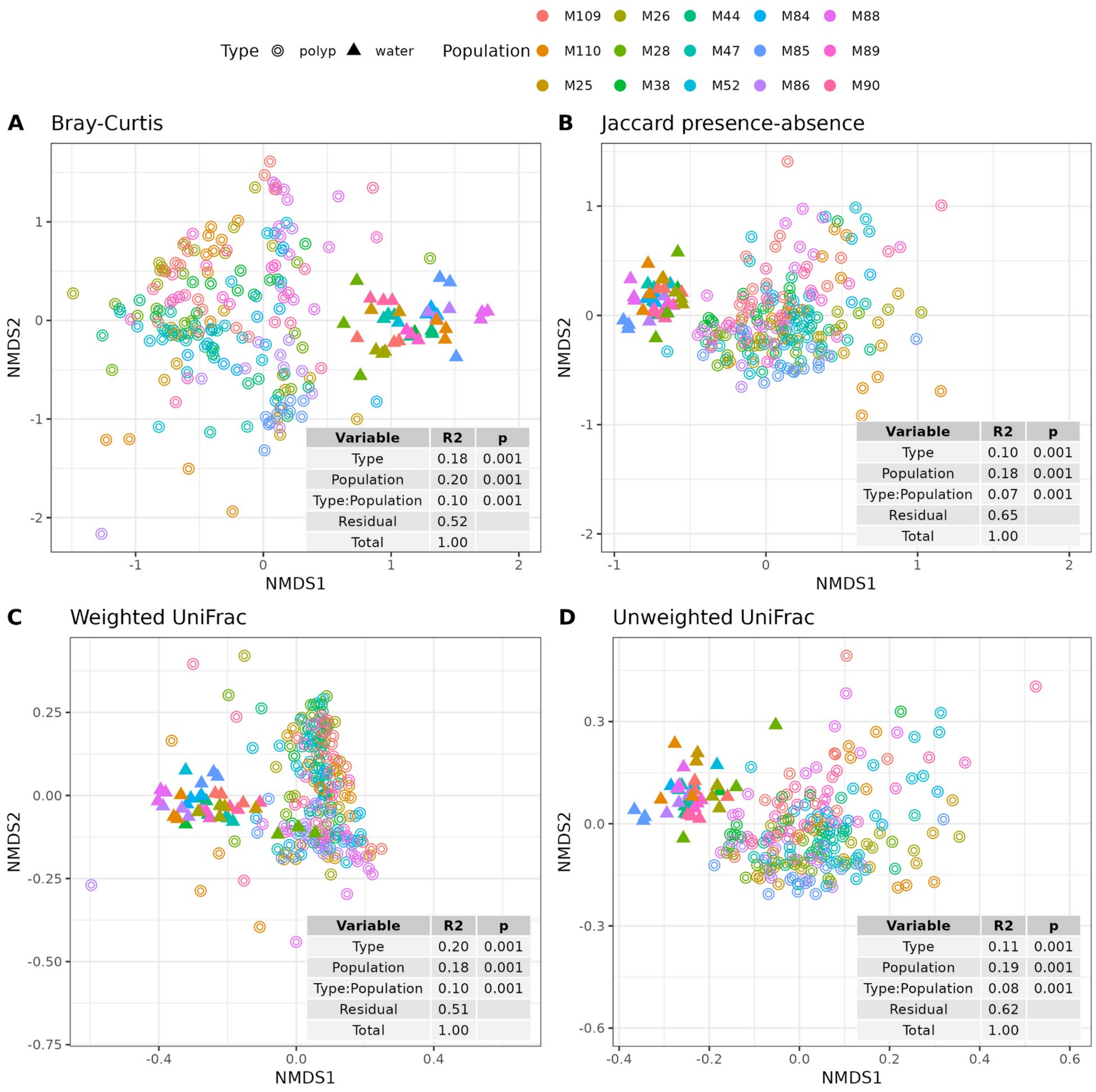

Supplementary Fig. 3 *Non-metric multidimensional scaling (NMDS) analysis based on Bray-Curtis distances of microbial communities found on Hydra polyps and matching environmental (water) samples based on four distinct beta-diversity metrics: Bray-Curtis index (A), Jaccard presence-absence index (B), weighted UniFrac (C) and unweighted UniFrac (D). Sample type (water vs. polyp), source population and their interaction had a significant effect on the microbiota differences using all four indices.*

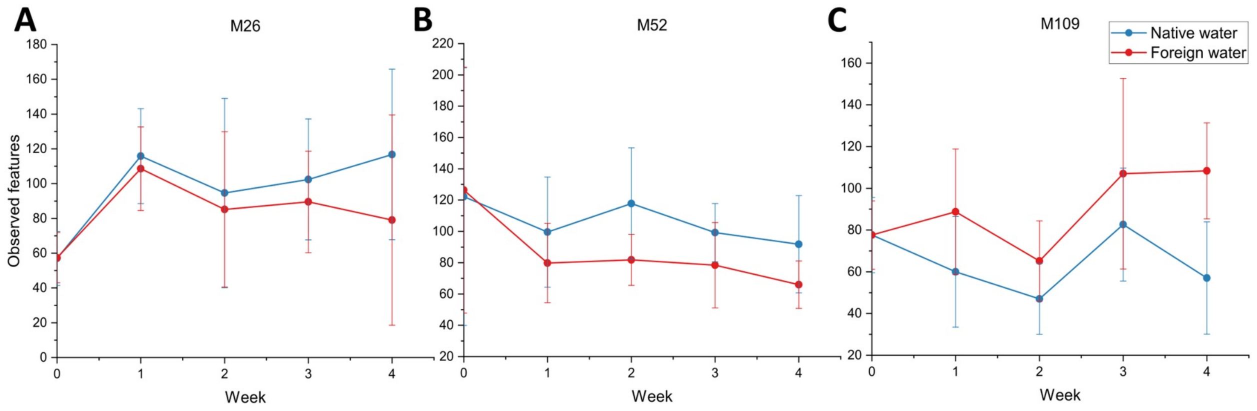

Supplementary figure 4: *Alpha diversity of the site-specific samples along the 4 weeks depending upon the source of cultivation water. The graphs represent the changes in Observed features for sites (A) M26, (B) M52 and (C) M109. There was no significant change in alpha diversity for all sites at any time point.*

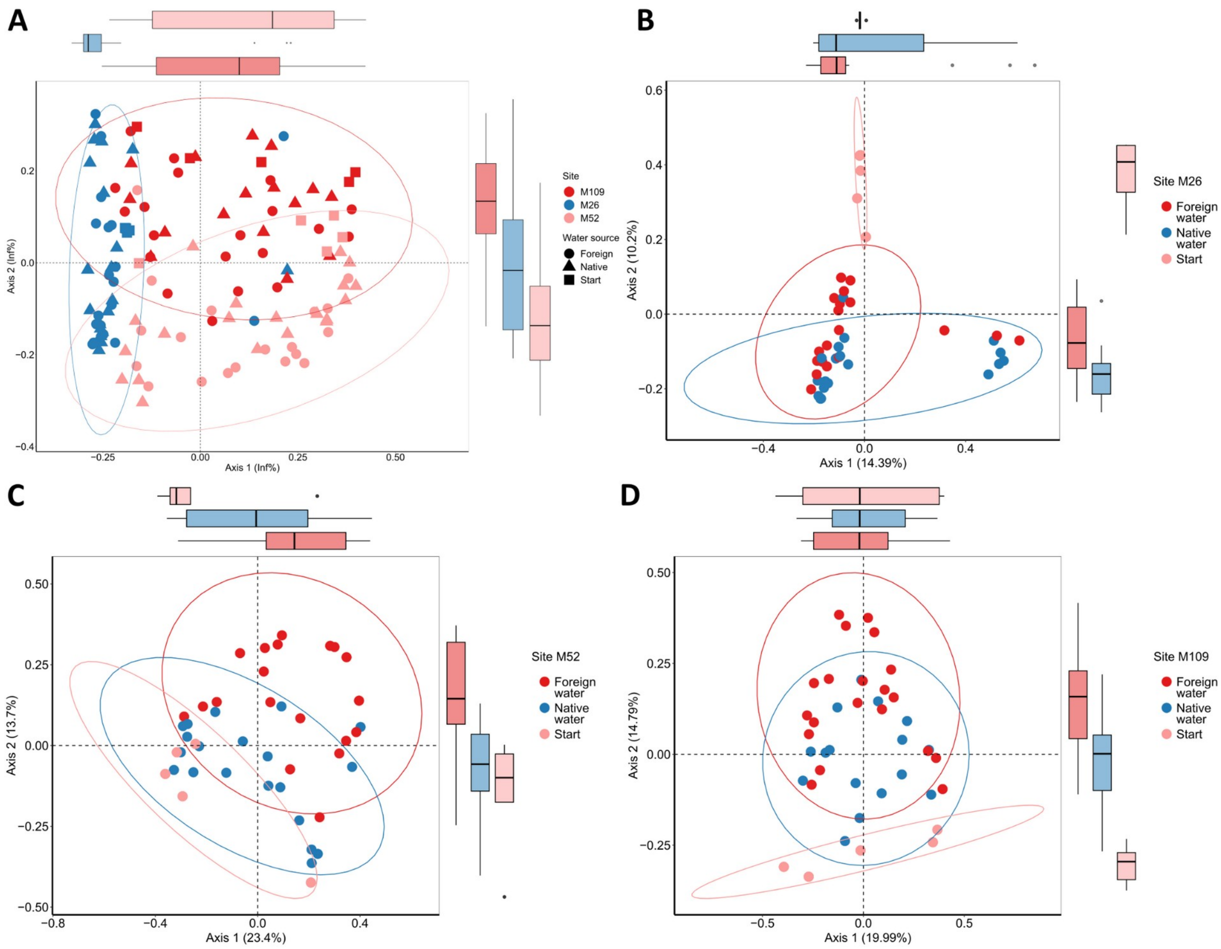

Supplementary figure. 5: *Beta diversity PCoA plot showing Jaccard distances of polyps from different sites that were maintained in the lab with water from 'native' or 'foreign' source. (A) The PCoA plot shows dissimilarity among the microbiome of polyps from the three sites (ADONIS, R2=0.123229, p=0.001) (B,C,D) There was a clear separation between the start samples and the samples receiving either of the waters cumulatively (Jaccard distances, PERMANOVA: M26- start vs native - adj.p < 0.05, start vs foreign - adj.p < 0.05; M52- start vs native - adj.p < 0.05, start vs foreign - adj.p < 0.05; M109- start vs native - adj.p < 0.05, start vs foreign - adj.p < 0.05)*

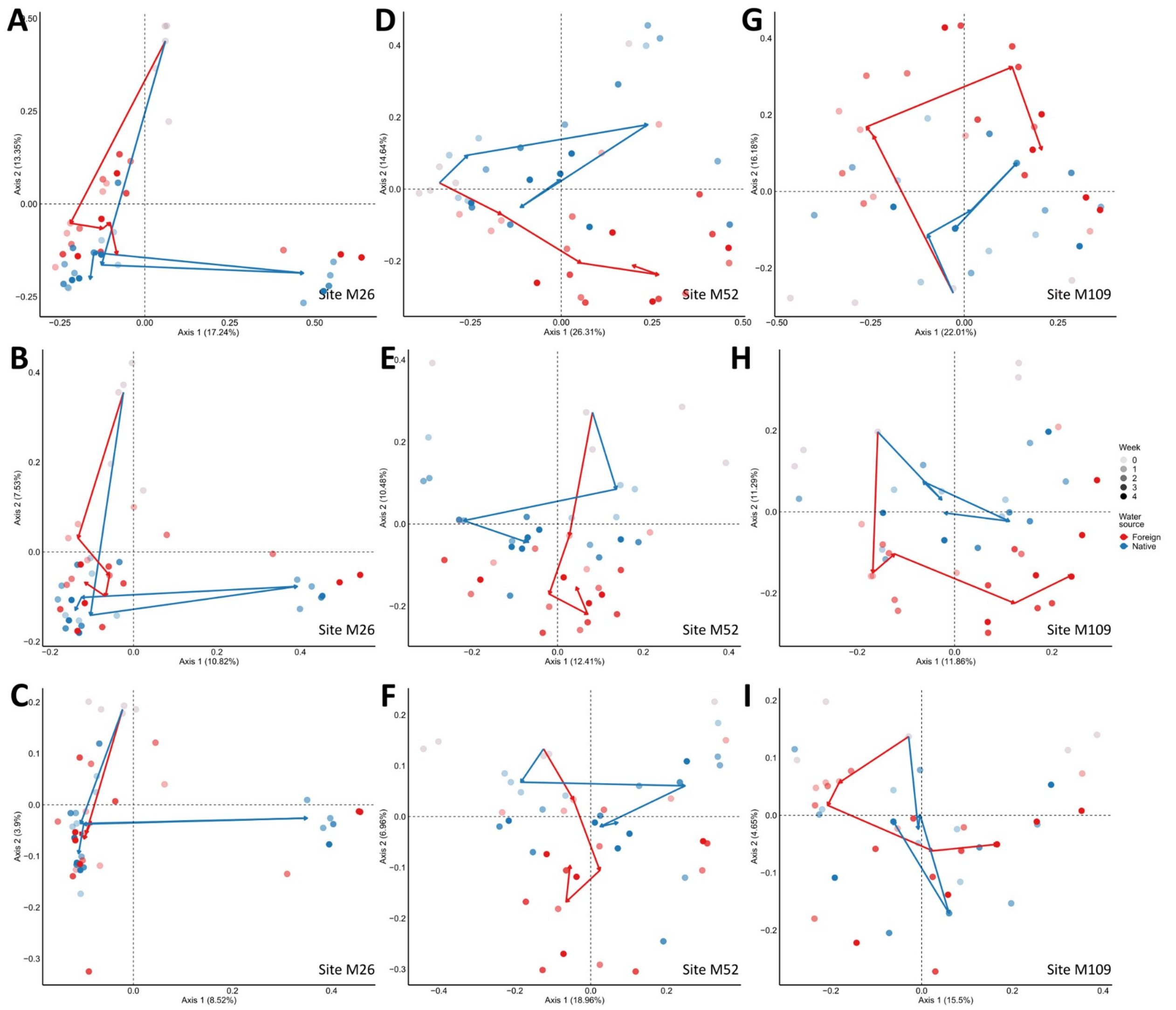

Supplementary figure 6: *The plots show beta diversity for Bray-Curtis (A,D,G) and Unweighted (B,D,F) UniFrac distances for polyps receiving water from native and foreign sources for the sites M26 (A,B,C), M52 (D,E,F) and M109 (G,H,I)*

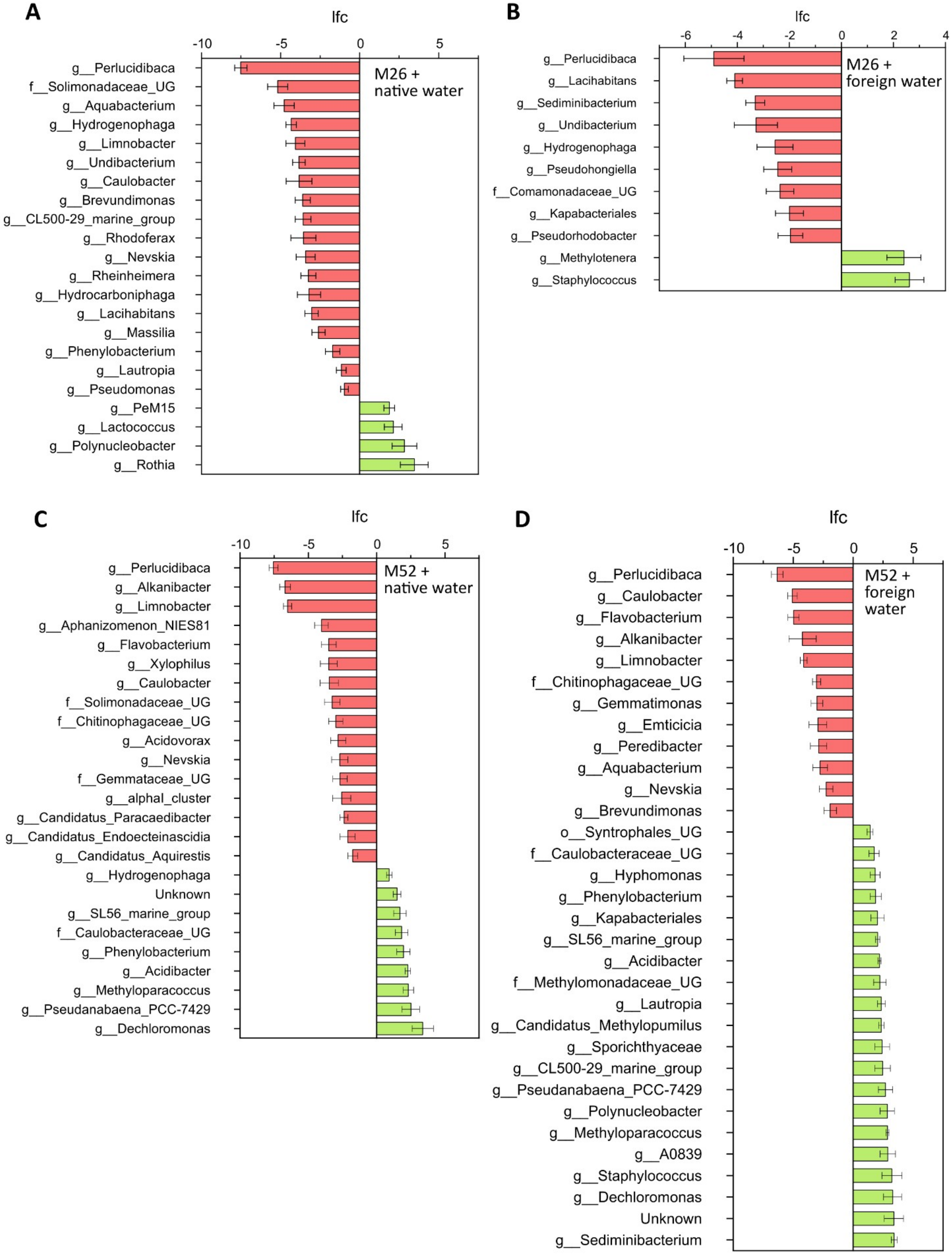

A
lfc
-10
-5
0
5
M26 + native water
g__Perlucidibaca
f__Solimonadaceae_UG
g__Aquabacterium
g__Hydrogenophaga
g__Limnobacter
g__Undibacterium
g__Caulobacter
g__Brevundimonas
g__CL500-29_marine_group
g__Rhodoferax
g__Nevskia
g__Rheinheimera
g__Hydrocarboniphaga
g__Lacihabitans
g__Massilia
g__Phenylobacterium
g__Lautropia
g__Pseudomonas
g__PeM15
g__Lactococcus
g__Polynucleobacter
g__Rothia
B
lfc
-6
-4
-2
0
2
4
M26 + foreign water
g__Perlucidibaca
g__Lacihabitans
g__Sediminibacterium
g__Undibacterium
g__Hydrogenophaga
g__Pseudohongiella
f__Comamonadaceae_UG
g__Kapabacteriales
g__Pseudorhodobacter
g__Methylotenera
g__Staphylococcus
C
lfc
-10
-5
0
5
M52 + native water
g__Perlucidibaca
g__Alkanibacter
g__Limnobacter
g__Aphanizomenon_NIES81
g__Flavobacterium
g__Xylophilus
g__Caulobacter
f__Solimonadaceae_UG
f__Chitinophagaceae_UG
g__Acidovorax
g__Nevskia
f__Gemmataceae_UG
g__alphaI_cluster
g__Candidatus_Paracaedibacter
g__Candidatus_Endoecteinascidia
g__Candidatus_Aquirestis
g__Hydrogenophaga
Unknown
g__SL56_marine_group
f__Caulobacteraceae_UG
g__Phenylobacterium
g__Acidibacter
g__Methyloparacoccus
g__Pseudanabaena_PCC-7429
g__Dechloromonas
D
lfc
-10
-5
0
5
M52 + foreign water
g__Perlucidibaca
g__Caulobacter
g__Flavobacterium
g__Alkanibacter
g__Limnobacter
f__Chitinophagaceae_UG
g__Gemmatimonas
g__Emticicia
g__Peredibacter
g__Aquabacterium
g__Nevskia
g__Brevundimonas
o__Syntrophales_UG
f__Caulobacteraceae_UG
g__Hyphomonas
g__Phenylobacterium
g__Kapabacteriales
g__SL56_marine_group
g__Acidibacter
f__Methylomonadaceae_UG
g__Lautropia
g__Candidatus_Methylopumilus
g__Sporichthyaceae
g__CL500-29_marine_group
g__Pseudanabaena_PCC-7429
g__Polynucleobacter
g__Methyloparacoccus
g__A0839
g__Staphylococcus
g__Dechloromonas
Unknown
g__Sediminibacterium

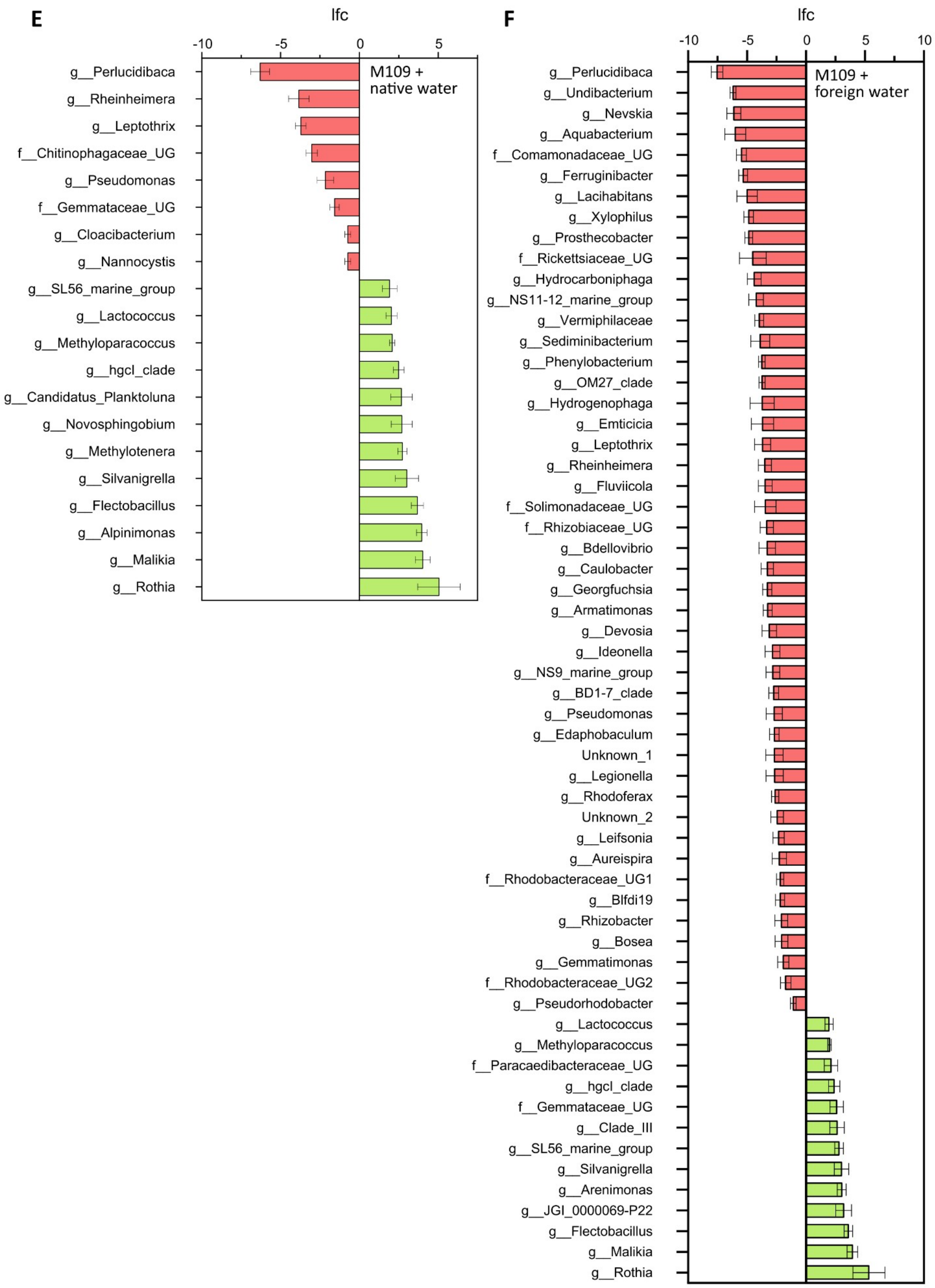
E
lfc
-10
-5
0
5
M109 + native water
g__Perlucidibaca
g__Rheinheimera
g__Leptothrix
f__Chitinophagaceae_UG
g__Pseudomonas
f__Gemmataceae_UG
g__Cloacibacterium
g__Nannocystis
g__SL56_marine_group
g__Lactococcus
g__Methyloparacoccus
g__hgcI_clade
g__Candidatus_Planktoluna
g__Novosphingobium
g__Methylotenera
g__Silvanigrella
g__Flectobacillus
g__Alpinimonas
g__Malikia
g__Rothia
F
lfc
-10
-5
0
5
10
M109 + foreign water
g__Perlucidibaca
g__Undibacterium
g__Nevskia
g__Aquabacterium
f__Comamonadaceae_UG
g__Ferruginibacter
g__Lacihabitans
g__Xylophilus
g__Prosthecobacter
f__Rickettsiaceae_UG
g__Hydrocarboniphaga
g__NS11-12_marine_group
g__Vermiphilaceae
g__Sediminibacterium
g__Phenylobacterium
g__OM27_clade
g__Hydrogenophaga
g__Emticicia
g__Leptothrix
g__Rheinheimera
g__Fluviicola
f__Solimonadaceae_UG
f__Rhizobiaceae_UG
g__Bdellovibrio
g__Caulobacter
g__Georgfuchsia
g__Armatimonas
g__Devosia
g__Ideonella
g__NS9_marine_group
g__BD1-7_clade
g__Pseudomonas
g__Edaphobaculum
Unknown_1
g__Legionella
g__Rhodoferax
Unknown_2
g__Leifsonia
g__Aureispira
f__Rhodobacteraceae_UG1
g__Blfdi19
g__Rhizobacter
g__Bosea
g__Gemmatimonas
f__Rhodobacteraceae_UG2
g__Pseudorhodobacter
g__Lactococcus
g__Methyloparacoccus
f__Paracaedibacteraceae_UG
g__hgcI_clade
f__Gemmataceae_UG
g__Clade_III
g__SL56_marine_group
g__Silvanigrella
g__Arenimonas
g__JGI_0000069-P22
g__Flectobacillus
g__Malikia
g__Rothia

Supplementary figure 7: *The figure represents the differential taxa for each site receiving water from native or foreign source. These taxa were identified using ANCOMBC analysis (alpha < 0.05, log2 fold change (lfc) > 1 or < -1). The comparisons represented here show the differential abundance of taxa between the start point and the 4 week-time point for each condition. Green color represents enrichment at the beginning of the experiment (start) and the red color represents the enrichment at the end (4-weeks).*

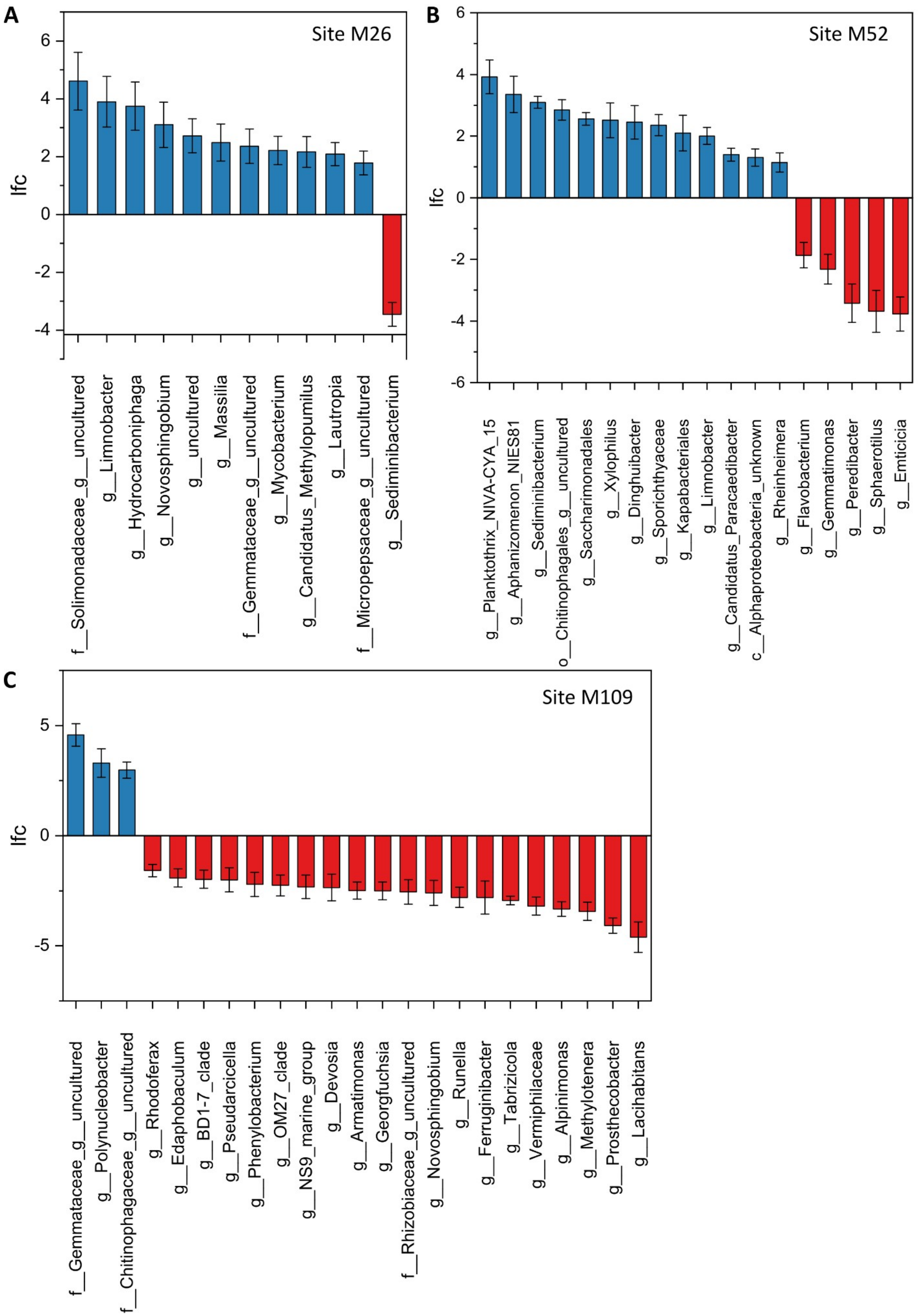

A
Site M26
lfc
6
4
2
0
-2
-4
f__Solimonadaceae_g__uncultured
g__Limnobacter
g__Hydrocarboniphaga
g__Novosphingobium
g__uncultured
g__Massilia
f__Gemmataceae_g__uncultured
g__Mycobacterium
g__Candidatus_Methylopumilus
g__Lautropia
f__Micropepsaceae_g__uncultured
g__Sediminibacterium
B
Site M52
lfc
6
4
2
0
-2
-4
-6
g__Planktothrix_NIVA-CYA_15
g__Aphanizomenon_NIES81
g__Sediminibacterium
o__Chitinophagales_g__uncultured
g__Saccharimonadales
g__Xylophilus
g__Dinghuibacter
g__Sporichthyaceae
g__Kapabacteriales
g__Limnobacter
g__Candidatus_Paracaedibacter
c__Alphaproteobacteria_unknown
g__Rheinheimera
g__Flavobacterium
g__Gemmatimonas
g__Peredibacter
g__Sphaerotilus
g__Emticicia
C
Site M109
lfc
5
0
-5
f__Gemmataceae_g__uncultured
g__Polynucleobacter
f__Chitinophagaceae_g__uncultured
g__Rhodoferax
g__Edaphobaculum
g__BD1-7_clade
g__Pseudarcicella
g__Phenylobacterium
g__OM27_clade
g__NS9_marine_group
g__Devosia
g__Armatimonas
g__Georgfuchsia
f__Rhizobiaceae_g__uncultured
g__Novosphingobium
g__Runella
g__Ferruginibacter
g__Tabrizicola
g__Vermiphilaceae
g__Alpinimonas
g__Methylotenera
g__Prosthecobacter
g__Lacihabitans

Supplementary figure 8: *The figure represents the ANCOMBC results (alpha < 0.05, log2 fold change (lfc) > 1 or < -1) for differential taxa abundance for all three sites at the 4 week time point comparing the animals receiving native water vs foreign water. The blue and red bars represent the taxa enriched in animals receiving native water and foreign water respectively.*

Supplementary tables:

Supplementary table 1: Field sampling sites, dates and habitat types.

Population ID	Locality	Coordinates	Sampling date	Habitat type
M25	Látókép	47.56112N; 21.45294E	9th Dec. 2020	Fishing lake
M26	Hortobágy	47.57600N; 21.14736E	9th Dec. 2020	Canalized river
M28	Tiszadorogma	47.67131N; 20.86631E	9th Dec. 2020	Oxbow lake
M38	Győrtelek	47.94814N; 22.43066E	25th Nov. 2020	Oxbow lake
M44	Tiszalúc	48.03713N; 21.09813E	4th Dec. 2020	Oxbow lake
M47	Muhi	47.98464N; 20.93812E	4th Dec. 2020	Fishing lake
M52	Apagy	47.98650N; 21.94818E	25th Nov. 2020	Fishing lake
M84	Fegyvernek	47.26131N; 20.51993E	30th Nov. 2020	Oxbow lake
M85	Szajol	47.17998N; 20.31345E	30th Nov. 2020	Oxbow lake
M86	Szolnok	47.14409N; 20.25968E	30th Nov. 2020	Oxbow lake
M88	Tiszakécske	46.92352N; 20.08092E	2nd Dec. 2020	Oxbow lake
M89	Lakitelek	46.85658N; 19.99109E	2nd Dec. 2020	Oxbow lake
M90	Tiszaalpár	46.82120N; 20.00168E	2nd Dec. 2020	Oxbow lake
M109	Tiszabábolna	47.68253N; 20.82136E	24th Nov. 2020	Oxbow lake
M110	Tiszadob	48.01004N; 21.17015E	4th Dec. 2020	Oxbow lake

Supplementary table 2: The table shows PERMANOVA results for beta-diversity comparisons among all sites for Bray-Curtis dissimilarity and Jaccard distance.

Group 1	Group 2	Bray-Curtis dissimilarity			Jaccard distance		
		pseudo-F	p-value	q-value	pseudo-F	p-value	q-value
M26	M109	6.489602	0.001	0.002	14.11601	0.001	0.0012
M26	M52	7.120385	0.001	0.002	15.0627	0.001	0.0012
M52	M109	9.802525	0.001	0.002	10.2278	0.001	0.0012

Supplementary table 3: The table shows PERMANOVA results for beta diversity comparisons for site M26 for Bray-Curtis dissimilarity between different groups and Jaccard distance between different groups. q-value represents the corrected p-value with Benjamini & Hochberg correction.

Site M26

Group 1	Group 2	Time	Bray-Curtis dissimilarity			Jaccard index		
			pseudo F	p-value	q-value	pseudo F	p-value	q-value
Start	Native water	week 1	3.976034	0.011	0.033	3.46983	0.007	0.014727
		week 2	2.868283	0.012	0.033231	2.023676	0.004	0.014727
		week 3	2.274463	0.015	0.036	2.49561	0.008	0.014727
		week 4	2.351733	0.026	0.052	2.41887	0.024	0.030857
Start	Foreign water	week 1	2.843162	0.005	0.029455	1.898068	0.01	0.015652
		week 2	1.672298	0.021	0.046588	1.871583	0.006	0.014727
		week 3	1.915696	0.029	0.054947	2.006727	0.025	0.031034
		week 4	1.990688	0.005	0.029455	1.933549	0.009	0.014727

Supplementary table 4: The table shows PERMANOVA results for beta diversity comparisons for site M52 for Bray-Curtis dissimilarity between different groups and Jaccard distance between different groups. q-value represents the corrected p-value with Benjamini & Hochberg correction.

Site M52

Group 1	Group 2	Time	Bray-Curtis dissimilarity			Jaccard index		
			pseudo F	p-value	q-value	pseudo F	p-value	q-value
Start	Native water	week 1	2.001799	0.074	0.093103	2.684868	0.008	0.014727
		week 2	5.114692	0.022	0.0495	3.071672	0.007	0.014727
		week 3	2.849804	0.025	0.052941	3.09343	0.008	0.014727
		week 4	2.352378	0.019	0.048	2.621416	0.009	0.014727
Start	Foreign water	week 1	1.866539	0.069	0.092	2.864428	0.002	0.014727
		week 2	5.383412	0.007	0.036	3.307075	0.009	0.014727
		week 3	4.50022	0.007	0.036	2.904962	0.009	0.014727
		week 4	3.916415	0.016	0.048	2.812641	0.016	0.018581

Supplementary table 5: The table shows PERMANOVA results for beta diversity comparisons for site M109 for Bray-Curtis dissimilarity between different groups and Jaccard distance between different groups. q-value represents the corrected p-value with Benjamini & Hochberg correction.

Site M109

Group 1	Group 2	Time	Bray-Curtis dissimilarity			Jaccard index		
			pseudo F	p-value	q-value	pseudo F	p-value	q-value
Start	Native water	week 1	2.095652	0.051	0.11	2.673718	0.005	0.024923
		week 2	0.959545	0.433	0.472364	2.580763	0.011	0.026
		week 3	3.147842	0.038	0.11	2.959907	0.017	0.027
		week 4	1.090584	0.363	0.421548	2.262768	0.026	0.032276
Start	Foreign water	week 1	2.638141	0.048	0.11	4.077944	0.015	0.027
		week 2	2.369141	0.032	0.104727	3.630186	0.009	0.024923
		week 3	4.088882	0.008	0.087429	4.082596	0.004	0.024923
		week 4	3.409158	0.011	0.087429	4.331836	0.007	0.024923
